# Furanone loaded aerogels are effective antibiofilm therapeutics in a model of chronic *Pseudomonas aeruginosa* wound infection

**DOI:** 10.1016/j.bioflm.2023.100128

**Published:** 2023-05-05

**Authors:** Chris R. Proctor, Megan G. Taggart, Barry M.G. O'Hagan, Paul A. McCarron, Ronan R. McCarthy, Nigel G. Ternan

**Affiliations:** a Nutrition Innovation Centre for Food and Health (NICHE), School of Biomedical Sciences, Ulster University, Northern Ireland, UK; b School of Pharmacy and Pharmaceutical Sciences, Ulster University, Northern Ireland, UK; c Division of Biosciences, Department of Life Sciences, College of Health and Life Sciences, Brunel University London, Uxbridge, UB8 3PH, UK; d Genomic Medicine Research Group, School of Biomedical Sciences, Ulster University, Northern Ireland, UK

## Abstract

Almost 80% of chronic wounds have a bacterial biofilm present. These wound biofilms are caused by a range of organisms and are often polymicrobial. *Pseudomonas aeruginosa* is one of the most common causative organisms in wound infections and readily forms biofilms in wounds. To coordinate this, *P. aeruginosa* uses a process known as quorum sensing. Structural homologues of the quorum sensing signalling molecules have been used to disrupt this communication and prevent biofilm formation by *Pseudomonas*. However, these compounds have not yet reached clinical use. Here, we report the production and characterisation of a lyophilised PVA aerogel for use in delivering furanones to wound biofilms. PVA aerogels successfully release a model antimicrobial and two naturally occurring furanones in an aqueous environment. Furanone loaded aerogels inhibited biofilm formation in *P. aeruginosa* by up to 98.80%. Further, furanone loaded aerogels successfully reduced total biomass of preformed biofilms. Treatment with a sotolon loaded aerogel yielded a 5.16 log reduction in viable biofilm bound cells in a novel model of chronic wound biofilm, equivalent to the current wound therapy Aquacel AG. These results highlight the potential utility of aerogels in drug delivery to infected wounds and supports the use of biofilm inhibitory compounds as wound therapeutics.

## 1 Introduction

*Pseudomonas aeruginosa* is a ubiquitous Gram-negative pathogen present in a wide range of environments [19,41] and it has become one of the most prevalent nosocomial infections in the developed world. The prevalence of multidrug resistance in this organism has increased greatly [24,43] and it is estimated that drug resistant *P. aeruginosa* was responsible for 84,600 deaths worldwide in 2019 alone [40]. In addition to its high frequency of antimicrobial resistance, *P. aeruginosa* has the ability to form biofilms on a range of biotic and abiotic surfaces [49]. These biofilms consist of a collection of bacterial cells encased in a self-produced polymer matrix, which provides several advantages for *P. aeruginosa* including protection from physical and mechanical stresses, host immune systems, and classical antibiotic treatments [30]. Biofilms have been implicated in a wide range of diseases including cystic fibrosis [9,29,56], infective endocarditis [11] and periodontal disease [53]. It is quite possible, however, that chronic wounds represent the most widespread biofilm-mediated disease [4,55,62,64]. Almost 80% of chronic wounds have an associated bacterial biofilm [36], and *P. aeruginosa* is one of the most common causative organisms [16,65]. During such infections, the organism grows almost exclusively in this form [5,42] resulting in an inflammatory state which delays the wound healing process. This inflammatory state is maintained long-term due to the highly persistent nature of the biofilm resulting in a significant delay in – and often total failure of – normal wound healing. It is clear, therefore, that any new wound therapy must address biofilm as a priority.

The regulation of bacterial biofilm formation is governed by a cell density dependent method of bacterial communication known as quorum sensing (QS). QS allows bacteria to coordinate complex behaviours and respond to various external stimuli or stresses [1]. Due to the role of QS in both biofilm formation and virulence factor production, the QS systems of *P. aeruginosa* represent attractive targets for novel therapies. One potentially useful approach has been the use of compounds with a high degree of structural similarity to the native QS signalling molecules. While many studies have focused on synthesising such novel molecules [28,38]*,* an alternative approach is to use naturally occurring structurally analogous compounds, such as furanones.

Furanones are a class of natural molecules found primarily in marine and terrestrial plants where they often act as flavour and aroma compounds. Both synthetic and natural furanones effectively inhibit QS-controlled behaviours in a range of pathogenic organisms including *Escherichia coli* [50,61,67] and *P. aeruginosa* [2,22,44,46,51,52]. Furanones have also shown efficacy in *in vivo* models of *Pseudomonas* infection, showing an ability to not only reduce QS by *P. aeruginosa*, but also to improve bacterial clearance from the murine lung [23,63]. For a comprehensive overview of furanones and their potential as QS inhibitors, the reader is referred to a recent review by the authors [48].

Despite promising results in the laboratory, QS inhibitors have not yet progressed beyond *in vivo* models. One of the main factors limiting their translation to clinical use is the lack of appropriate and effective delivery method. Considering the role of QS in biofilm development, the impact of biofilm formation on chronic wound persistence, and the significant need for new methods of delivering QS inhibitors to chronic wounds, the aim of this work is to develop a novel PVA aerogel for use as a method to deliver two naturally occurring furanones; furaneol and sotolon (Supplementary Fig. S1) to *P. aeruginosa* biofilms. These compounds have both previously been shown to have biofilm inhibitory activity at sub growth inhibitory concentrations (with previously reported MICs of 80.05 μg mL^−1^ for furaneol and an MIC of 200 μg mL^−1^ for sotolon) [[2], [3]]. Due to their previously demonstrated antibiofilm activity, these compounds were chosen for this study to show that they be delivered an retain their biological activity. Having shown the biological effect of these compounds we aim to assess the compounds’ efficacy when delivered to bacterial biofilms in a novel model of chronic wound biofilm with a polymer aerogel wound dressing material.

## 2 Methods

### 2.1 Chemicals and bacterial strain

All chemicals were purchased from Sigma Aldrich (Gillingham, UK) and used without further modification unless stated. All bacterial culture media were purchased from Oxoid (Hampshire, UK) and used according to the manufacturer's instructions.

Bacterial strain DSM50071 (Merck Sharp and Dohme) was purchased from Deutchse Sammlung von Mikrooganismen und Zellkulturen (DSMZ) (Braunschweig, Germany).

### 2.2 Preparation of minimally crosslinked poly (vinyl alcohol) aerogels

PVA aerogels were prepared by lyophilisation of PVA solutions of varying concentrations in deionised water. PVA solutions were prepared using Mw 31,000–50,000, 98–99% hydrolysed PVA. Two grams of PVA solution was pipetted into each well of a 12-well tissue culture plate (Sarstedt, Leicester, UK) which was then frozen at −30 °C for 6 h. Frozen samples were then placed, uncovered, in a freeze dryer (Labconco, USA) at 0.04 mBar and −80 °C for 24 h. Once dry, aerogel samples were removed from the 12 well plate and stored in an airtight container.

Drug-loaded PVA aerogels were prepared using this method by dissolving an appropriate amount of antibiotic in the PVA solution prior to aliquoting, freezing, and drying as above.

A full schematic for the preparation of the aerogels can be seen in Supplementary Fig. S2.

### 2.3 Electron microscopy analysis

Aerogel structure was assessed using a FEI Quanta™ 200 (FEI Company, Eindhoven, Netherlands) scanning electron microscope (SEM). Aerogels were divided into quarters for SEM analysis of both the surface morphology and the internal structure simultaneously. Samples were mounted on aluminium stubs and sputter coated using a gold/palladium target using a E5100 sputter coater (Polaron Equipment Ltd) and imaged with a beam acceleration of 30 kV. Images were acquired via the integrated imaging software (xT microscope control) and charge coupled device camera.

### 2.4 Micro CT analysis

Aerogels were prepared as described above but in a 96 well plate using 100 mg aliquots of PVA solution in the wells of a 96 well tissue culture plate giving aerogels with a 5 mm diameter and 5 mm depth. Aerogels were then fixed to the top of a metal spindle using 3M super 77 spray adhesive.

Samples were imaged at the Henry Mosely X-ray Imaging facility, at Manchester University using a Zeiss Xradia 520 Versa MicroCT scanner with a power of 70 kV at 71 μA. The sample was positioned 11.55 mm from the x-ray source and 9.55 mm from the x-ray detector. Each 1 mm^3^ area within the samples had 1600 individual projections taken. These projections were then stacked using ImageJ (v1.52). ImageJ was then used to compile a 3-dimensional projection of the scan. A representative 3D projection of each 1 mm^3^ sample was compiled.

### 2.5 Release studies

Drug loaded aerogels were placed on a wire mesh support structure so that the bottom surface of the aerogel was in contact with the surface of an aqueous receiver phase. Aliquots (500 μL) of the receiver phase were removed at 0, 10, 20, 30, 60, 120, 180, 240, 300, and 390 min and concentrations determined using UV–Visible spectrophotometry at 275 nm for ciprofloxacin or 285 nm for each furanone. Fresh deionised water was added at each time point to maintain the receiver phase volume.

### 2.6 Time-kill assay

Stationary phase overnight cultures of *P. aeruginosa* DSM50071 were diluted 1:100 in 50 mL sterile TSB and grown to an OD_600_ of 0.1 in a 200 mL Erlenmeyer flask. A PVA aerogel loaded with 64.7 μg of ciprofloxacin (an antimicrobial dose of ciprofloxacin as determined during MIC experiments and calculated based on the 50 mL volume of the culture to be treated and the total percentage drug release measured during release studies) was added to the bacterial culture which was incubated at 37 °C and 200 rpm. Aliquots of 100 μL were removed from the culture at 0, 1, 2, 3, 4, 5, 6, and 24 h post-treatment and viable cells enumerated via serial dilution and plating. Experiments with an appropriate vehicle control were also conducted.

### 2.7 Minimum inhibitory concentration

The minimum bactericidal concentrations (MBC) for furaneol, sotolon and ciprofloxacin against *P. aeruginosa* DSM50071 were determined using broth microdilution and incubation at 37 °C for 18 h followed by plating samples of each well on nutrient agar. The MBC was defined as the lowest concentration of furanone to result in no growth on nutrient agar after incubation at 37 °C for 18 h. To confirm the streak plate results a triphenyl tetrazolium chloride (TTC) assay was used. A 100 μL aliquot of each well of the MIC plate was transferred to a fresh 96-well plate and 100 μL of sterile 0.1% TTC in deionised waster was added. Each well was thoroughly mixed by repeated pipetting. The plate was incubated in the dark at 37 °C for 30 min. A change in colour from colourless to red indicated the presence of metabolically active cells.

### 2.8 Assessment of furanone treatment on established biofilm biomass

Biofilms were prepared in 12-well tissue culture plates. Each well was inoculated with 2 mL of a 1:100 dilution of a stationary phase overnight culture of *P. aeruginosa* DSM50071in tryptone soy broth (TSB). To assess the effect of early treatment on biofilm formation with each furanone 12-well plates were inoculated as described, and a sub-inhibitory dose (half MIC) of the furanone of interest was added to each well at the point of inoculation. Biofilms were then incubated in a static incubator at 37 °C for 24, 48, or 72 h. After treatment, remaining culture medium was removed from each well and the biofilms washed three times with deionised water to remove unbound cells and excess biofilm matrix. Biofilms were then stained with 2 mL of 0.1% (w/v) crystal violet solution and incubated at room temperature for 10 min. The crystal violet was removed and plates were washed in deionised water five times. Biofilms were destained with 30:70 acetic acid/water solution for 10 min following which resolubilised crystal violet was quantified spectrophotometrically at 570 nm.

To assess the impact of furanone treatment on established biofilm biomass, biofilms were grown for either 24 or 48 h prior to treatment. Biofilms were then processed and stained as described above.

### 2.9 Fluorescence imaging of furanone treated biofilm morphology

Biofilms for fluorescence microscopy were grown directly on glass slides. Sterile slides were placed in 15 mL of a 1:100 dilution of a stationary phase *P. aeruginosa* DSM50071 overnight culture. Cultures were either treated immediately with furanone and biofilms allowed to form for 24 h, or biofilms were grown for 24 h prior to furanone treatment followed by incubation for a further 24 h. Slides were then removed from liquid medium, planktonic cells washed off by submerging in sterile deionised water three times at which point the biofilms were allowed to air dry. Biofilms were stained with BacLight Live/Dead stain (Thermo Fisher, USA). A working BacLight reagent was prepared as per manufacturer's instructions. Stain was added to each slide so that the entire surface was covered. Stained substrates were incubated for 30 min in the dark at room temperature and then rinsed by gently submerging in sterile deionised water. Substrates were then allowed to air dry in the dark. Biofilms were visualised at x200 magnification using a Leica DM4000 microscope. Images were captured using the native Las AF software. Heatmaps of relative fluorescence intensity representations were prepared as previously described [45] using the ImageJ (v1.53) “3D Interactive Surface Plot” plugin (v 2.4).

### 2.10 *In vitro* assessment of aerogel delivered furanone against DSM50071

Biofilms were prepared in 12-well tissue culture plates as described above. To assess the inhibition of biofilm formation, furanone loaded aerogels were added to the 2 mL of culture either at the point of inoculation and biofilms were incubated with treatment for 24 h prior to quantification with crystal violet. To assess the effect of aerogel delivered furanone on preformed biofilms, biofilm pates were prepared and incubated at 37 °C for 24 h. Pre-formed biofilms were then treated by adding furanone loaded aerogels to the wells and incubated for a further 24 h prior to quantification with crystal violet.

### 2.11 Preparation of a novel *in vitro* wound model for the assessment of wound therapeutics

A novel semi-synthetic wound medium was produced using porcine myocyte lysate (PML), simulated body fluid (SBF), bovine serum albumin (BSA) (Sigma-Aldrich, Poole, UK) and laked horse blood. To produce the PML, 100 g of porcine muscle tissue was processed in a commercial blender (Kenwood, UK) with 150 mL of deionised water for 3 min until a homogenous mixture was formed. The mixture was then centrifuged at 17,500 rcf for 5 min following which the supernatant was recovered and gravity filtered to remove any remaining solids. BSA was added to the PML to a final concentration of 56 mg mL^−1^, followed by filter sterilisation using a 0.2 μm syringe filter. This solution was then warmed to 40 °C. The simulated wound bed medium (SWBM) was prepared by combining 30 mL of warm PML/BSA solution, 10 mL of sterile 5x concentration SBF solution (0.68 M sodium chloride, 27 mM potassium chloride, 1.25 mM sodium phosphate dibasic, 2.2 mM potassium phosphate dibasic trihydrate, 6.5 mM calcium chloride, 5 mM magnesium sulphate, 21 mM sodium bicarbonate, and 27.5 mM glucose in deionised water [27]), 7.5 mL of molten 5% agar solution and 2.5 mL of laked horse blood. Molten SWBM (10 mL) was poured into sterile Teflon lined containers (KitchenCraft, Birmingham, UK) measuring 75 mm × 45 mm and stored at 4 °C overnight to allow complete gelation of the medium. Wound beds were removed aseptically, trimmed to 30 × 30 mm and transferred to stainless steel mesh platforms. Sterile mesh platforms with simulated wound beds were placed in a sterile plastic container and a 20 mL aliquot of sterile 1x simulated body fluid added, ensuring that the surface of the liquid was in contact with the bottom of the gel wound bed. A sterile polycarbonate membrane was placed on the surface of the wound bed and inoculated with 5 μL of a 1:100 dilution of stationary phase overnight cultures of *P. aeruginosa* DSM50071. Inoculated wound beds were incubated at 37 °C to allow biofilms to reach 1 × 10^6^ CFU/biofilm (approximately 6 h). Pieces of each wound dressing or furanone loaded aerogel were then applied to the surface of the biofilms and incubated for a further 24 h to simulate a typical wound dressing cycle. Treatments were then removed and biofilms were transferred to 10 mL of sterile PBS and biofilm bound cells resuspended by mechanical agitation with a sterile plastic inoculation loop for 15 s followed by three separate 10 s periods of vortex mixing. Resuspended biofilm samples were serially diluted to 10^−11^ with plating on nutrient agar for enumeration of viable cells. This was performed using three biological replicates and data expressed as the average total number of viable cells per biofilm.

## 3 Results

### 3.1 Minimally crosslinked poly (vinyl alcohol) aerogels are prepared simply via a one-step method

Lyophilised aerogels are a potentially useful pharmaceutical material for the delivery of active pharmaceutical compounds to infected wounds. However, minimal changes in material formulation have the potential to alter an aerogel's ability to release compounds and absorb liquid thus greatly impacting its utility as a drug delivery device. To assess the effects of polymer density on aerogel structure, aerogels were prepared using varying concentrations of PVA solution. All aerogels had a similar opaque white appearance and displayed a range of physical properties; those produced using higher concentration polymer solutions (10% w/w and 7.5% w/w) were firmer, and those produced using lower polymer concentrations (5% w/w) were more sponge-like. Aerogels produced using a 1% w/w PVA solution had a very soft, fluffy texture similar to cotton wool (Supplementary Fig. S3).

**Fig. 1:**
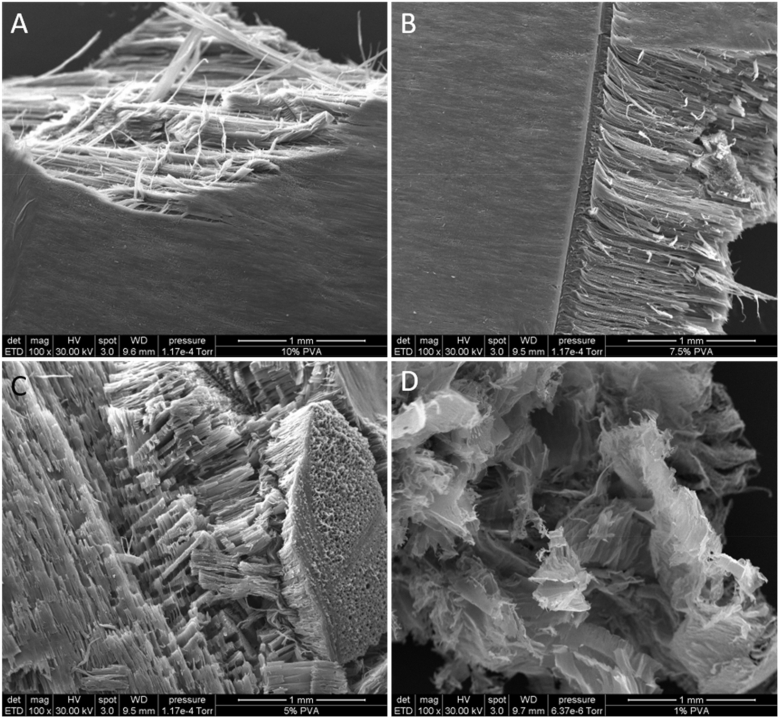
Scanning electron micrographs of aerogels with varying PVA concentration. (a) 10% PVA aerogel (b) 7.5% PVA aerogel (c) 5% PVA aerogel (d) 1% PVA. High levels of fibre organisation are seen in the higher concentration PVA gels with lower fibre organisation in the lower concentration PVA gels.

To gain further insight into the structure of the material, the microscale structure of the aerogels was characterised using SEM. Aerogels made using 10% PVA solutions exhibited a fibrous but highly ordered internal structure which showed uniaxial alignment of the fibres (Fig. 1a). A similar structure was also observed in aerogels produced using a 7.5% PVA solution (Fig. 1b). In contrast, aerogels produced using a 5% PVA solution displayed an internal structure that appeared to be an intermediate between that of a lamellar structure and a fibrous structure. The fibres in these aerogels showed areas of localised order in fibre direction but a relative disorder across the whole sample (Fig. 1c). The cotton-like aerogels (1% PVA) had no obvious internal order, appearing instead as thin sheets of material (Fig. 1d).

**Fig. 2:**
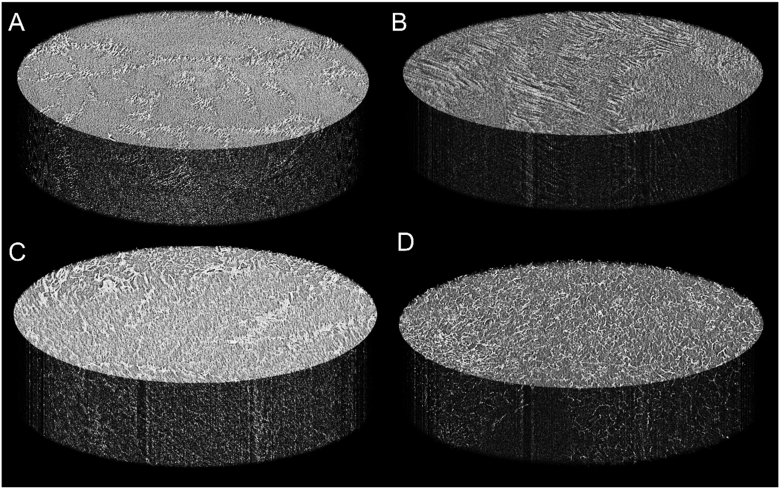
Representative, three-dimensional, reconstructions of micro-CT images of the developed aerogels. Light coloured portions of the material indicate a higher density. Aerogels prepared with 10% PVA solution (A) show areas of more tightly packed fibres and large areas of more even density suggesting a high a degree of structural heterogeneity within the gel. Aerogels made with 7.5% PVA (B) exhibited similar structure to 10% aerogels. Areas of varying material density were seen in 5% aerogels (C) and 1% aerogels (D) though a greater overall level of homogeneity was apparent in these. Images are representative of 3 independent replicates.

Aerogels are a diffusion-based drug release system and are, therefore, dependent on rehydration. As the homogeneity of the internal structure of the aerogels could impact on material rehydration and subsequent drug release, micro-CT analysis was used to assess internal structure. The 10% aerogel was partially homogeneous but had veins of material with differing density throughout the structure. These areas of altered density suggested that, within them, the packing of the fibre stands in the gel was less ordered. Micro-CT imaging of the 7.5% aerogel revealed similar structure to the 10% PVA gel, with areas of highly homogenous density and also areas exhibiting variable density. These areas of heterogeneity in the 7.5% PVA aerogel were less numerous than those in the 10% PVA aerogel, but were larger in area. Continuing the observed trend, the 5% PVA aerogel possessed significantly more heterogeneity: areas of higher density, and, therefore, tightly packed fibres, were considerably smaller and more evenly distributed throughout the sample than those observed in the 10% and 7.5% PVA aerogels (Fig. 2 a-c). The micro-CT analysis of the 1% PVA aerogel revealed that it was significantly less dense than the other samples, with fewer, smaller sized, areas of higher density material being evenly distributed throughout the sample (Fig. 2 d).

### 3.2 Aerogels effectively release an antimicrobial payload

**Fig. 3:**
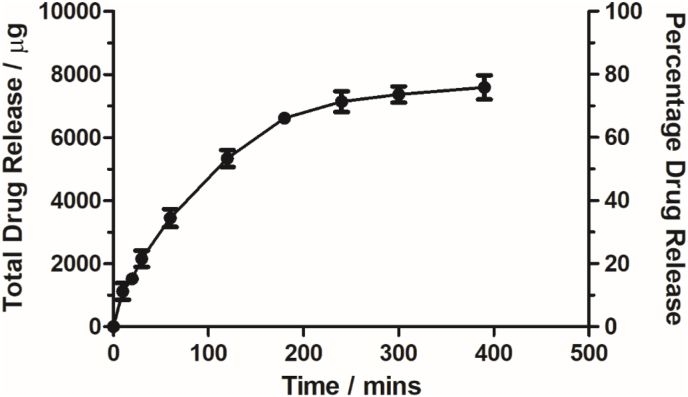
Ciprofloxacin release from aerogels made using 10% PVA solution. A maximum release of 7.59 mg was released from the aerogel over 390 min. This represents 75.9% release of the total loaded drug volume. Ciprofloxacin was released with clear first order kinetics. N = 3, values represents the mean of three independent replicates ± S.D.

In order to demonstrate the utility of PVA aerogels as a drug release system, aerogels prepared using 10% PVA were loaded with the model antimicrobial ciprofloxacin. Ciprofloxacin was released in a highly controlled manner with apparent first order kinetics, suggesting that the aerogels are indeed a diffusion-based drug delivery system. A total drug release of 7.59 mg (76% of the total loaded drug) over 390 min was achieved with no obvious burst release at early time points indicating that release rate was dependent on the concentration of drug remaining in the gel [6] (Fig. 3).

**Fig. 4:**
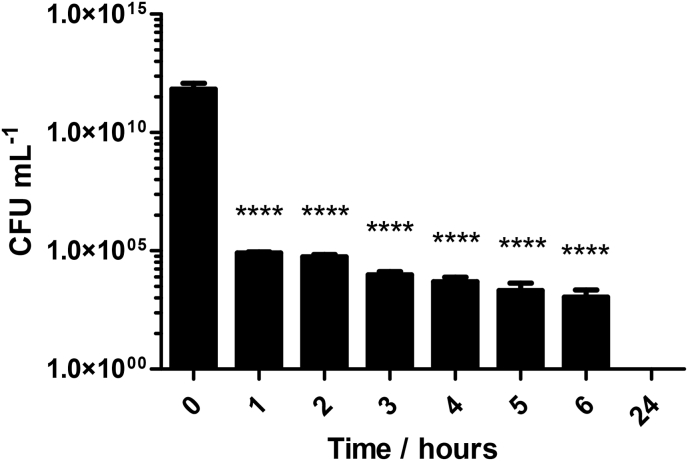
Time dependent killing of planktonic *P.aeruginosa* cells by ciprofloxacin-loaded PVA aerogels. Following the application of treatments, a 6.7 log reduction in viable cells (p < 0.0001). Bacterial killing continued for the duration of the experiment and resulted in total bacterial killing after 24 h of treatment. **** indicates p < 0.0001. N = 3, values represents the mean of three independent replicates ± S.D. Analysis was by one-way ANOVA with a post-hoc Dunnett's multiple comparison test.

Having established that ciprofloxacin was released from loaded aerogels, the bactericidal potential of the released ciprofloxacin was assessed. Cell viability over time was assessed using planktonic cultures of DSM50071. The addition of ciprofloxacin-loaded aerogels to liquid cultures of DSM50071 led to rapid and effective killing of bacterial cells, with a 6.7 log reduction in viable cells observed at 1 h (p = <0.0001) and a subsequent decrease in viable cell numbers each additional hour. Following the full 24 h treatment, total bacterial cell killing was achieved (p = < 0.0001) (Fig. 4). Unloaded control aerogels showed no significant killing.

Having proved that an antimicrobial loaded aerogel could effectively release payload and kill bacterial cells we aimed to show efficacy of furanone loaded aerogels against DSM50071.

### 3.3 Furaneol and sotolon strongly inhibit biofilm formation by *P. aeruginosa* DMS50071

The minimum inhibitory concentration of both furaneol and sotolon against DSM50071 were assessed and found to be 8 mg mL^−1^ and 8.65 mg mL^−1^ respectively (Supplementary Fig. S4). To first confirm that furaneol and sotolon showed activity against DSM50071, the antibiofilm effects of sub-inhibitory concentrations of each furanone was assessed over 72 h. When treated with a sub-inhibitory dose of furaneol (4 mg mL^−1^), consistent reductions in biofilm biomass were seen when compared with an untreated control. Reductions of 76.67%, 77.02% and 88.33% were seen at 24 h, 48 h and 72 h respectively (Supplementary Fig. 5). When biofilms were grown in the presence of a sub-inhibitory dose of sotolon (4.33 mg mL^−1^) reductions in biofilm biomass of 87.20%, 86.58% and 66.13% were observed at 24 h, 48 h and 72 h, respectively (Supplementary Fig. S5).

### 3.4 Furaneol and sotolon significantly reduce *P. aeruginosa* DMS50071 biofilm biomass in mature biofilms

Chronic wounds often develop biofilm rapidly and therefore treatments must seek to eradicate established biofilm, rather than simply preventing its development. Thus, we assessed the effect of furanone treatment on established biofilms.

**Fig. 5:**
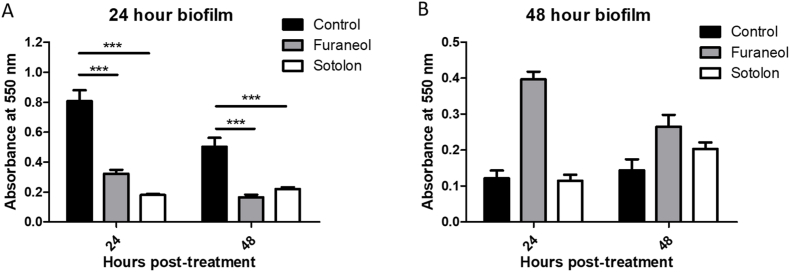
The effect of furanone treatment on total biofilm biomass of established biofilms. When used to treat a 24 h old biofilm (A) 4 mg mL^−1^ furaneol resulted in a considerable decrease in biofilm biomass (60.01% and 67.13% after 24 h and 48 h of treatment, respectively). When used to treat a 48 h old biofilm (B) a similar dose of furaneol resulted in significant increases in biofilm biomass. When used to treat a 24 h old biofilm (A) 4.33 mg mL^−1^ sotolon resulted in 77.66% and 56.12% decreases in total biofilm biomass. When used to treat a 48 h old biofilm (B), sotolon had no significant effect. Data shown represents the mean of three independent replicates (±S.D.). Analysis was by independent t-tests. ***p = ≤ 0.001.

When treated with 4 mg mL^−1^ furaneol, 24 h old biofilm biomass decreased by 60.01% after 24 h of treatment and 67.13% after 48 h treatment as measured by crystal violet staining (Fig. 5a). When treated with a 4 mg mL^−1^ concentration of furaneol, 48 h old biofilm biomass increased by 226% after 24 h of treatment, but showed no significant difference compared to untreated controls after 48 h of treatment (Fig. 5b). When treated with 4.33 mg mL^−1^ of sotolon, a biofilm established for 24 h exhibited a reduction in biomass of 77.66% and 56.12% at 24 h and 48 h post-treatment, respectively (Fig. 5a). When treated with the same dose of sotolon, 48 h old biofilms exhibited no significant changes in biofilm biomass (Fig. 5b).

Having shown useful reductions in biofilm biomass following a 24 h treatment with furanones, cell viability staining was then used to show the impact of furanone treatment of established biofilm morphology. Control biofilms stained with BacLight were densely populated with SYTO9 stained (green) stained cells, with extensive coverage of the substrate (Supplementary Figure S6 A-C). When treated with 4 mg mL^−1^ furaneol after 24 h of growth very sparsely populated biofilms developed with observable areas of higher density that appeared to be clumped cells (Supplementary Figure S6 D-F). When treated with 4.33 mg mL^−1^ sotolon biofilms were again equally sparse and exhibited a similar clumped morphology. We observed however that, when treated with sotolon, the clumps of cells generally appeared smaller (Supplementary Figure S6 G-I). This sparse appearance of the furanone-treated biofilms, without a corresponding increase in non-viable biofilm-bound cells, suggests a degree of biofilm dispersal caused by furanone treatment.

**Fig. 6:**
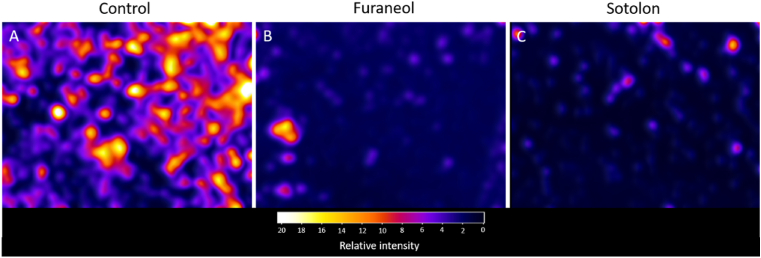
Mapping fluorescence intensity of the previously obtained Baclight images showed that untreated biofilms (A) had a clustered morphology with high cell density in a large proportion of the clusters. When treated with furaneol (B) and sotolon (C) significant reductions in fluorescence intensity were observed. This is indicative of a reduction in SYTO9 stained cells within the biofilm and, thus, a reduction in the overall live cell density of the biofilm. Images are representative of three independent replicates.

Baclight images of live biofilm bound cells were converted to relative fluorescence intensity using the ImageJ “3D Interactive Surface Plot” plugin to further demonstrate the change in biofilm morphology. Treatment with both furanones resulted in a considerable reduction in SYTO9 stained cells and overall biofilm population density (Fig. 6A–C).

### 3.5 Furanones are effectively released from PVA aerogels

To be useful as a wound therapeutic, PVA aerogels must be able to effectively release furanones. Furaneol showed controlled first order release from a 10% PVA aerogel, result in in release of 70.75% of the total loaded drug over 300 min (Supplementary Fig. S7). Conversely, release of sotolon from a PVA aerogel exhibited initial burst release, with a total of 58.35% of the total loaded drug being released in 30 min, reaching a final release of 93.85% of total loaded drug after 300 min (Supplementary Fig. S7).

### 3.6 Aerogel delivered furanone can effectively inhibit biofilm formation and reduce biofilm biomass *in vitro*

**Fig. 7:**
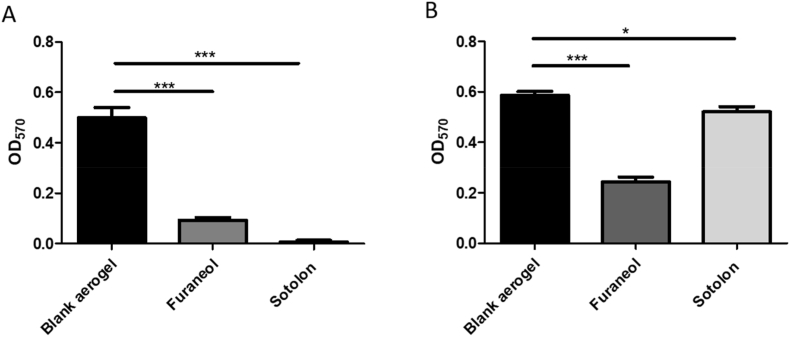
The effects of aerogel delivered furanones on (A) forming biofilms and (B) preformed biofilms. Both furaneol and sotolon effectively inhibited the formation of biofilm by DSM50071 with reductions of 81.56 and 98.80% respectively. When used to treat preformed biofilms (B) furaneol gave a 58.53% reduction while sotolon yielded only an 11.12% reduction. Data shown represents the mean of three independent replicates (±S.D.). Analysis was by independent t-tests. *p=<0.05 ***p = ≤ 0.001.

To ensure aerogel delivered furanone retains its efficacy, furanone loaded aerogels were used to treat preformed biofilms and applied to cultures immediately after inoculation. Both furaneol and sotolon were able to significantly inhibit the formation of biofilm when added at the point of inoculation at sub inhibitory concentrations using a PVA aerogel yielding an 81.56% and 98.80% decrease, respectively (Fig. 7a). Furthermore, both compounds were able to reduce the biomass of an established biofilm, with furaneol (58.53% reduction) being significantly more effective than sotolon (11.12% reduction) (Fig. 7b).

### 3.7 Sotolon loaded aerogels are equally or more effective than currently used antimicrobial wound dressings

In this work, we have shown that both furaneol and sotolon are potent inhibitors of biofilm formation in *P. aeruginosa* DSM50071 and that both of these compounds are able to reduce biofilm biomass in established biofilms when delivered using a PVA aerogel. To be considered as a potential wound therapy, however, antimicrobial efficacy for furanone loaded aerogels must be assessed in comparison to current clinical therapies.

**Fig. 8:**
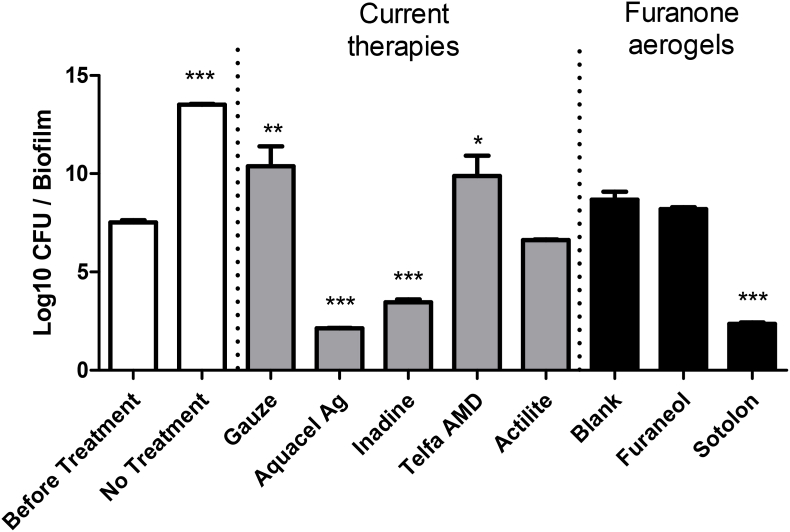
Changes in viable cell numbers following treatment with clinically used wound dressings using a novel *in vitro* wound model. Treatment with gauze caused cells to proliferate with a 2.87 log increase after 24 h. Aquacel Ag resulted in a 5.39 log reduction. Inadine treatment showed a 4.06 log decrease in viable biofilm bound cells. Telfa AMD had no bactericidal effect and biofilms proliferated during treatment with a 2.36 log increase. Treatment with Actilite showed no significant change in viable cell numbers. Both unloaded and furaneol loaded aerogels showed no significant difference in viable biofilm bound cells while sotolon aerogel treatment yielded a 5.16 log redction. Data shown represents the mean of three independent replicates ± S.D. Analysis is by one-way ANOVA of the log10 values compared to the before treatment control. *p = 0.05, **p = 0.01, ***p = 0.001.

To assess this effectiveness, biofilms were grown in our novel wound model consisting of a semi-synthetic, wound-like growth medium. As bacterial resistance to antimicrobials is significantly impacted by nutrient availability and nutrient composition [25,54] the wound-like nutrient profile used in this model gives rise to a biofilm that is more representative of the clinical scenario than other *in vitro* biofilms. Biofilms were treated with a range of current clinically used antimicrobial wound therapies. Two interventions, gauze (no active ingredient) and Telfa AMD (containing polyhexamethylene biguanide) showed no ability to reduce viable biofilm bound cells and, indeed biofilms treated with these continued to proliferate over the course of the treatment (2.87 and 2.36 log increase respectively). Actilite, a therapy containing manuka honey, resulted in no reduction in biofilm albeit with no proliferation of bacterial numbers above the baseline. Use of dressings containing two of the most commonly used active ingredients for infected wounds, Aquacel Ag and Inadine (containing silver and povidone iodine respectively) [8] resulted in a significant reduction in viable biofilm bound cells: 5.39 log for Aquacel Ag, and 4.06 log for Inadine. Unloaded aerogels did not result in a significant reduction in biofilm cells. Despite its effectiveness in planktonic cell culture, furaneol loaded aerogels resulted in no significant reduction or proliferation of biofilm bound cells however the application of sotolon-loaded aerogels resulted in a 5.16 log reduction in viable cells, making sotolon loaded aerogels as effective as the current standard treatment, Aquacel Ag (Fig. 8).

## 4 Discussion

In this work we describe a PVA aerogel material and its potential to deliver two naturally occurring furanones as antibiofilm compounds against *P. aeruginosa* wound infection.

We developed and characterised a minimally crosslinked PVA aerogel for the controlled delivery of antimicrobial compounds. The developed material consists of a polymer solution that was frozen and lyophilised to yield a low-density material that would rehydrate in the presence of fluid and release an antimicrobial payload. While we did not use any crosslinking agent we hypothesise that the formation of crystallites (areas of high crystallinity) during the freezing process resulted in a small degree of crosslinking through hydrogen bonding, thus giving a minimally crosslinked, easily rehydrated system [20,26,47].

The microscale structure of the aerogel was examined under SEM and a degree of regularity in the internal structure, which reduced with decreasing polymer density, was apparent. It was unexpected that higher polymer concentrations showed greater structural order. Zhang *et al*. observed a similar alignment in freeze dried PVA solutions subjected to highly controlled directional freezing [68]. They suggested that uniaxial alignment of PVA strands was caused by phase separation during the freezing process [68]. The apparent alignment observed in our work may also occur through freeze concentration. Briefly, as solvent crystals form on cooling, a solute is forced out of the freezing solvent resulting in a higher concentration of solute at the boundary between the frozen and non-frozen solvent, thus concentrating the solute in the liquid phase (Butler, 2001, 2002). Here, we hypothesise that freeze concentration of the 10% polymer solution resulted in greater concentrations of PVA at the interface, consequently forcing the strands into alignment. The assessment of the impact of strand alignment on drug release kinetics merits significant investigation and was, therefore, beyond the scope of this work. The internal structures of the aerogels were then assessed by micro-CT. It was found that, in contrast to the SEM analysis the internal structure of the 10% PVA aerogel was the least broadly homogeneous, with large areas of significantly more dense material irregularly dispersed throughout. Internal structural homogeneity increased with decreasing polymer concentration.

Having developed aerogels, we assessed their potential as antimicrobial delivery systems. The release profile of ciprofloxacin from our aerogels showed controlled release of the payload, similar to previously reported drug release profiles of lyophilised aerogel-like materials [34]. It has been suggested that differences in the internal structure and structure stability of an aerogel can greatly impact the release of a loaded drug [60,69] and this lends weight to our hypothesis that, due to the unique internal structure of the aerogels developed in this work, these materials would be expected to release a payload differently to materials produced using methods such as critical point drying. Conversely, the similar release profiles observed in this work and other studies such as the work of Ma *et al.* [34] may be explained by the similar methods of aerogel production employed, which could yield materials with similar internal structures.

Aerogel delivery of ciprofloxacin to planktonic cultures of *P. aeruginosa* resulted in total bacterial kill at 24 h, in line with the observations of other researchers [10,32,33]. However, we quantitated bacterial killing over time, in contrast to a single 18–24 h endpoint assessment or zone of inhibition data [18,34,66]. In contrast to a number of published reports, our data provides suitable evidence to support the clinical use of the material. For example, if an antimicrobial aerogel was to be used in a wound dressing application, it is vital that not only can adequate bacterial killing can be achieved, but that the antimicrobial effect is rapid in its onset and sustained for the duration of the application [31]. While many studies fail to clearly show this, our data demonstrates these principles with a significant decrease in viable cell numbers in just 1 h, progressing to total killing within the time frame of a normal clinical wound dressing cycle.

The antibiofilm effect of furaneol and sotolon was then assessed at a concentration of half the established MIC over 72 h when administered at the point of inoculation. Both furaneol and sotolon showed high levels of biofilm inhibition. Furaneol treatment resulted in reductions in biofilm biomass of up to 88%. In 2014 Choi *et al*. showed that at concentrations as low as 12.81 ng mL^−1^ furaneol reduced biofilm biomass by up to 84.8% in furanone sensitive strains [2]. While the reductions reported in this work were similar to those reported by Choi *et al.,* the MIC and biofilm inhibitory concentrations of each furanone used here was several orders of magnitude greater due to the increased efflux capacity of DSM50071. This is likely due to the absence of several key genes in the DSM50071 genome, namely *mexR* and *nalC*, which encode known repressors of the MexAB-oprM efflux transporter [35], and *MdrR1* and *MdrR2*, which encode repressors of the EmrAB efflux pump [21]. The absence of these genes suggest that DSM50071 has a greatly increased capacity for drug efflux and, indeed, it has been shown that the absence of *mexR* and *nalC* in particular increases efflux of furanone compounds in *Pseudomonas aeruginosa* [14,15,35]. Although the strain used in this work was likely furanone resistant the data presented here clearly demonstrates that biofilm inhibition can still be achieved, non-lethally, in furanone resistant organisms with increased doses. Similar to furaneol, the biofilm inhibition of sotolon reported in this work is significantly higher than previously reported. Aldawsai et al. reported a maximum inhibition of approximately 60% following treatment with 50 μg mL-1 sotolon [3].

The effect of furanone treatment on biofilm morphology was assessed using BacLight staining. It was shown that when compared to untreated biofilms, those grown in the presence of furaneol showed a high degree of clumping with aggregates of low cell density being apparent at 24 h. Biofilms grown in the presence of sotolon, again, appeared as poorly populated, clumped biofilms. These results were corroborated with fluorescence intensity mapping of the obtained Baclight images. These results were expected as several studies have shown that interference with QS signalling using compounds such as usinic acid and can lead to the formation of biofilms with altered morphology [7,13].

Furaneol treatment of a 24 h old biofilm resulted in reductions up to 67.13% while furanone treatment showed no effect on 48 h biofilms. This may be due to poor penetration of the compound into the pre-formed biofilm, leading to poorer disruption of QS. It has been previously reported that, with antimicrobials such as vancomycin, as little as 20% of the administered dose is able to penetrate a bacterial biofilm [12]. If this also the case for furanones and *P. aeruginosa* biofilms, it may be that biofilm bound cells are receiving lower doses of furanone than anticipated. The lack of observed effect against a 48 h biofilm is likely due to a shift in the transcriptional profile of the biofilm. It has been shown that at 48 h quorum QS in *P. aeruginosa* gene expression shifts from processes such as motility and surface attachment which are known to be QS mediated to processes such as chemotaxis. This shift away from QS mediated behaviour is likely the reason for the diminished effect of furanones in 48 h biofilms [58,59].

In order to show the potential of furanones as a wound therapy it was necessary to show their ability to be loaded into and released from a pharmaceutically relevant controlled drug release system. Both furaneol and sotolon showed favourable release profiles from the aerogel materials, with the release of furaneol being more controlled than that of sotolon. This is similar to previous reports of drug release from aerogel materials, many of which show rapid burst release effects of drugs such as triflusal, paracetamol, nicotinic acid, and ketoprofen [17,37,39,57].

Finally, when assessed using an *in vitro* wound model, treatment of biofilms with sotolon loaded aerogels showed a significant reduction in biofilm bound cells. Following treatment, a 5.16 log decrease in biofilm bound cells was observed. This reduction in viable, biofilm-bound, cells was equivalent to the clinically used antimicrobial wound dressing Aquacel Ag which achieved a 5.39 log reduction in viable biofilm bound cells, and superior to Inadine, a povidone iodine based wound dressing. This indicates that sotolon loaded aerogels are a potentially viable option for the reduction of biofilm bound viable bacterial cells in chronic wounds infected with *P. aeruginosa*.

Taken together, our data presented here clearly demonstrates the potential utility of both furaneol and sotolon in the inhibition of the formation of new biofilm and the reduction of biomass in established biofilm. Furthermore, this work has shown that these compounds can easily be incorporated into a pharmaceutically relevant controlled release system and delivered to clinically relevant biofilms and retain their efficacy. While many studies show that the exogenous addition of furanones has antibiofilm effects, this work is the first to show that these compounds are viable active pharmaceutical ingredients which may be easily incorporated into novel wound therapeutics.

## Funding

RRMC is supported by a Biotechnology and Biological Sciences Research Council New Investigator Award BB/V007823/1. RRMC and CP are supported by the 10.13039/501100000691Academy of Medical Sciences/the 10.13039/100010269Wellcome Trust/the Government Department of Business, Energy and Industrial Strategy/the 10.13039/501100000274British Heart Foundation/Diabetes UK Springboard Award [SBF006\1040].

## CRediT authorship contribution statement

N.T., C.P., M.T., P.MC., and R.M.C designed the study. C.P., M.T., B.OH. conducted experimental work. C.P. performed data analysis. All authors contributed to the writing or editing o the manuscript.

## Declaration of competing interest

The authors have no competing interests to declare.

## Data Availability

Data will be made available on request.
