# Supplementary material for: Furanone loaded aerogels are effective antibiofilm therapeutics in a model of chronic Pseudomonas aeruginosa wound infection
Source: Biofilm. 2023 May 5;5:100128. doi: 10.1016/j.bioflm.2023.100128 (PMC10200818; doi:10.1016/j.bioflm.2023.100128)
Supplement: Multimedia component 1 [file mmc1.docx]

*Furanone loaded aerogels are effective antibiofilm therapeutics in a model of chronic Pseudomonas aeruginosa wound infection*

*(Supplementary Data)*

*
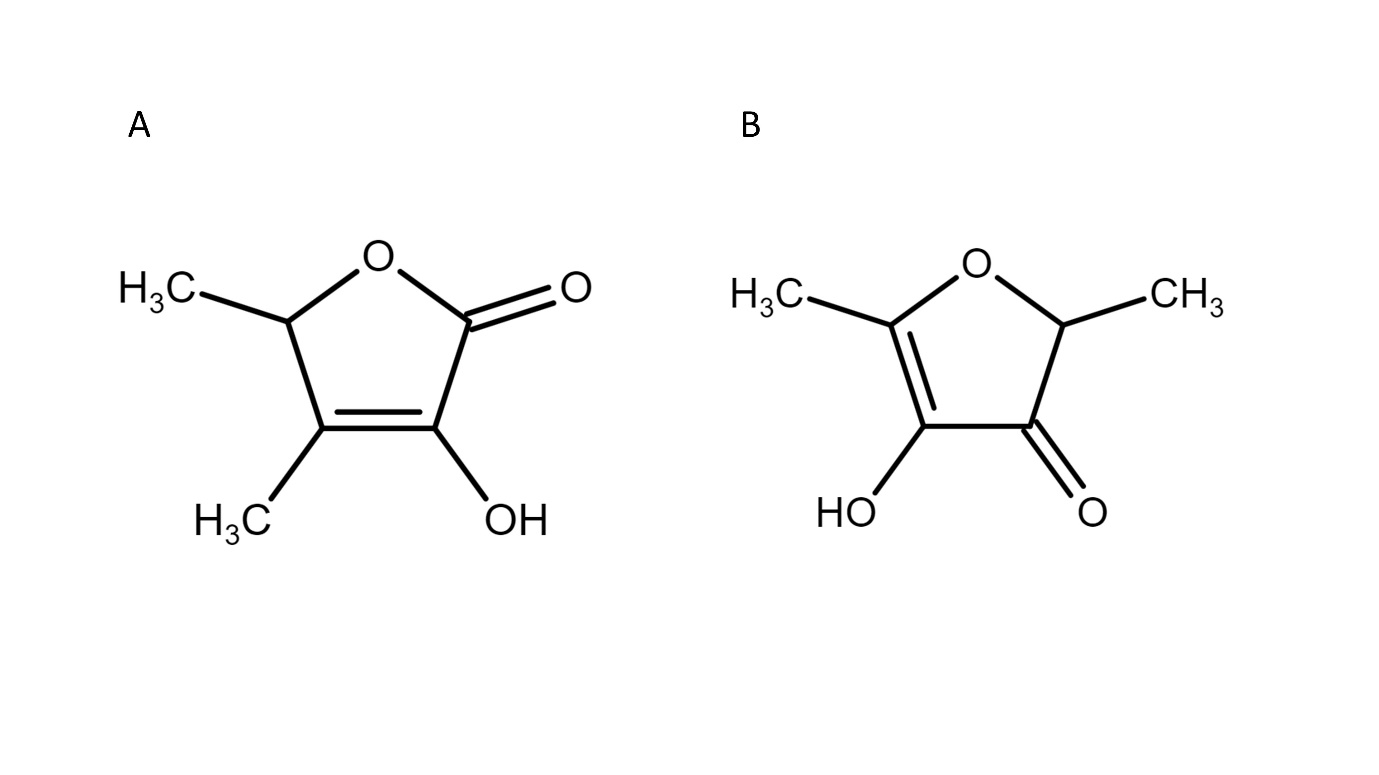
*

Supplementary Figure S1 - The chemical structure of the two naturally occurring furanones investigated in this study; (A) sotolon and (B) furaneol.


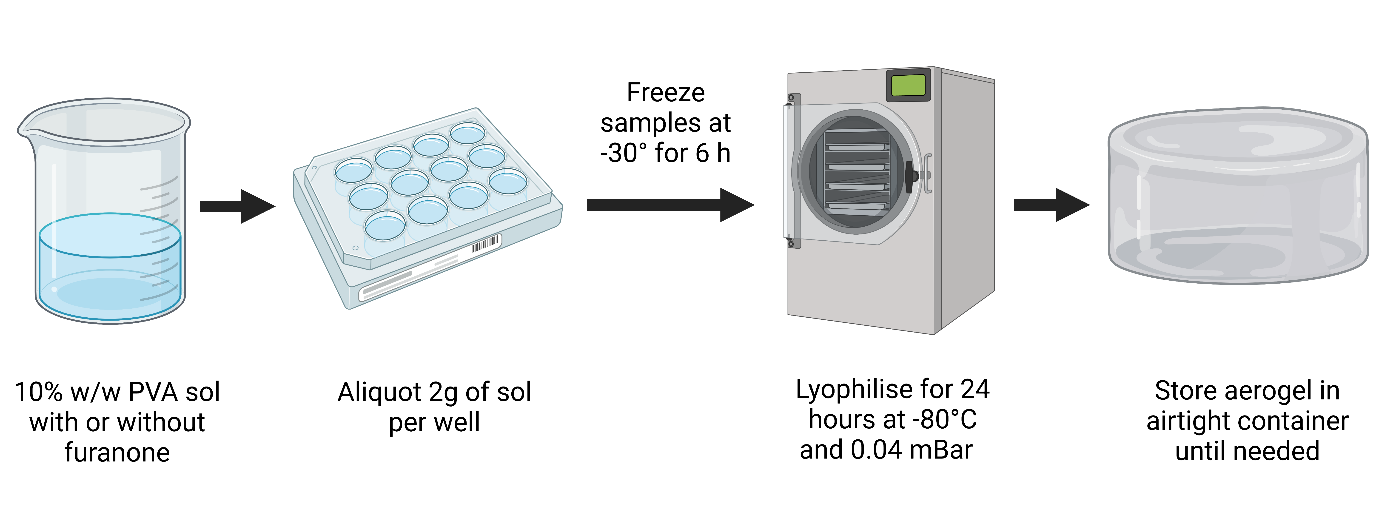


Supplementary Figure S2 - A schematic representation for the simple preparation of furanone loaded PVA aerogel materials


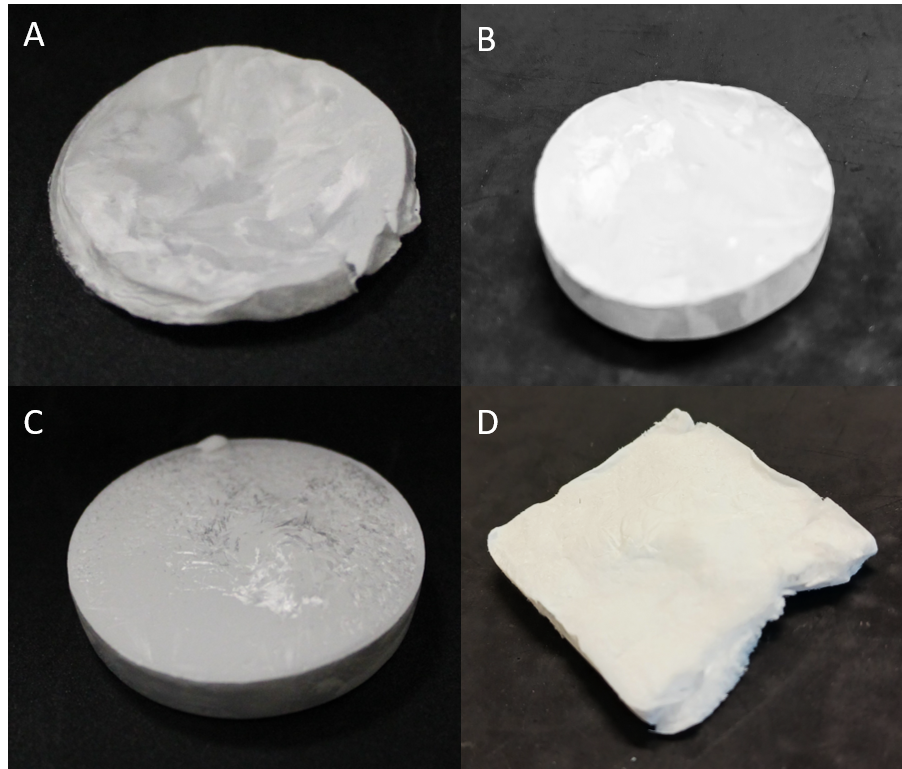


Supplementary Figure S3 – Macroscopic images of the PVA aerogel materials. Aerogels made using(A) 10% and (B) 7.5% PVA were firm but slightly pliable. Aerogels prepared using (C) 5% PVA were soft and easily compressed. Aerogels produced using (D) 1% PVA were extremely soft with a fluffy texture. They were very easily torn and compressed.


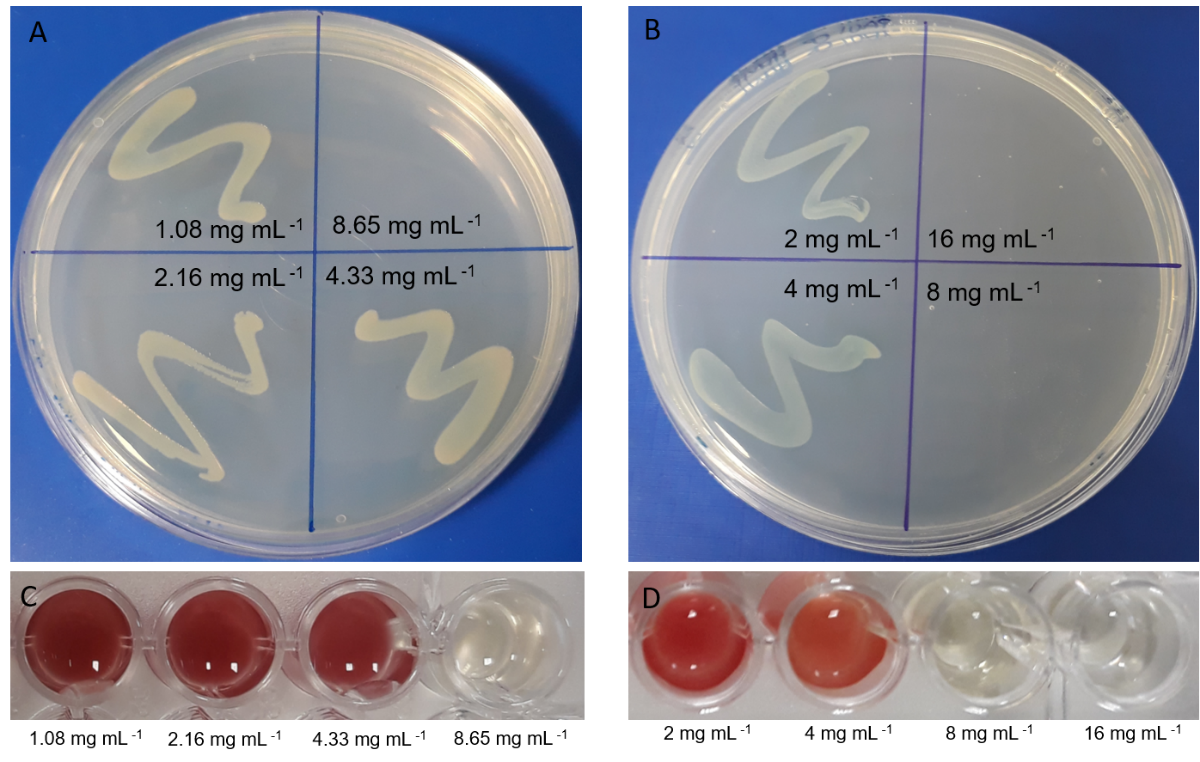


*Supplementary Figure S4 - Determination of the minimum inhibitory concentration (MIC) of each furanone using a streak plate method and a 2,3,5 triphenyl tetrazolium chloride (TTC) assay. The streak plate method showed that P. aeruginosa DSM50071 was effectively killed when treated with (A) 8.65 mg mL-1 of sotolon and (B) 8 mg mL-1 of HDMF. These results were confirmed using a TTC assay (C -D). Lack of a colour change to red indicated a lack of metabolically active cells in the sample. All results shown are representative images of 3 independent replicates.*


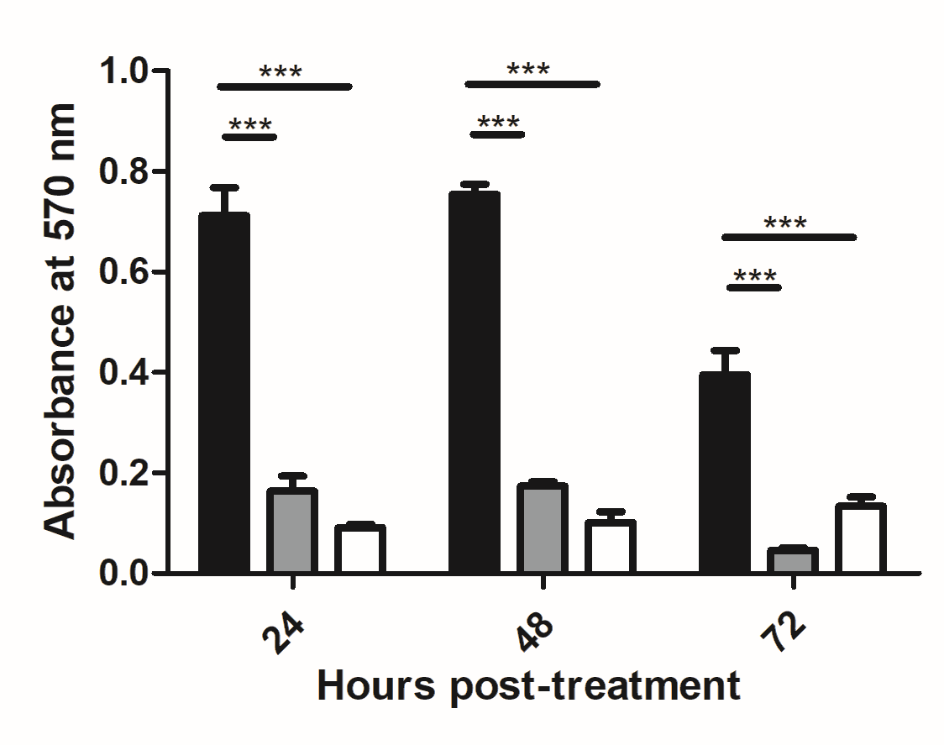


Supplementary Figure S5 – Total biofilm biomass of biofilms grown in the presence of furaneol and sotolon. It was shown that when biofilms were grown in the presence of 4 mg mL^-1^ furaneol (grey) biofilm biomass was reduced by 76.76%, 77.02% and 88.33% at 24 h, 48 h, and 72 h respectively when compared to untreated controls (black). Biofilms grown in the presence of 4.33 mg mL^-1^ sotolon (white) reductions of 87.20%, 86.58% and 66.13% were seen at 24 h, 48 h, and 72 h respectively. Data shown represents the mean of three independent replicates (± S.D.). Analysis was by two-way ANOVA with a Bonferroni post-hoc test to compare all treatments to untreated controls. *** p = ≤ 0.001


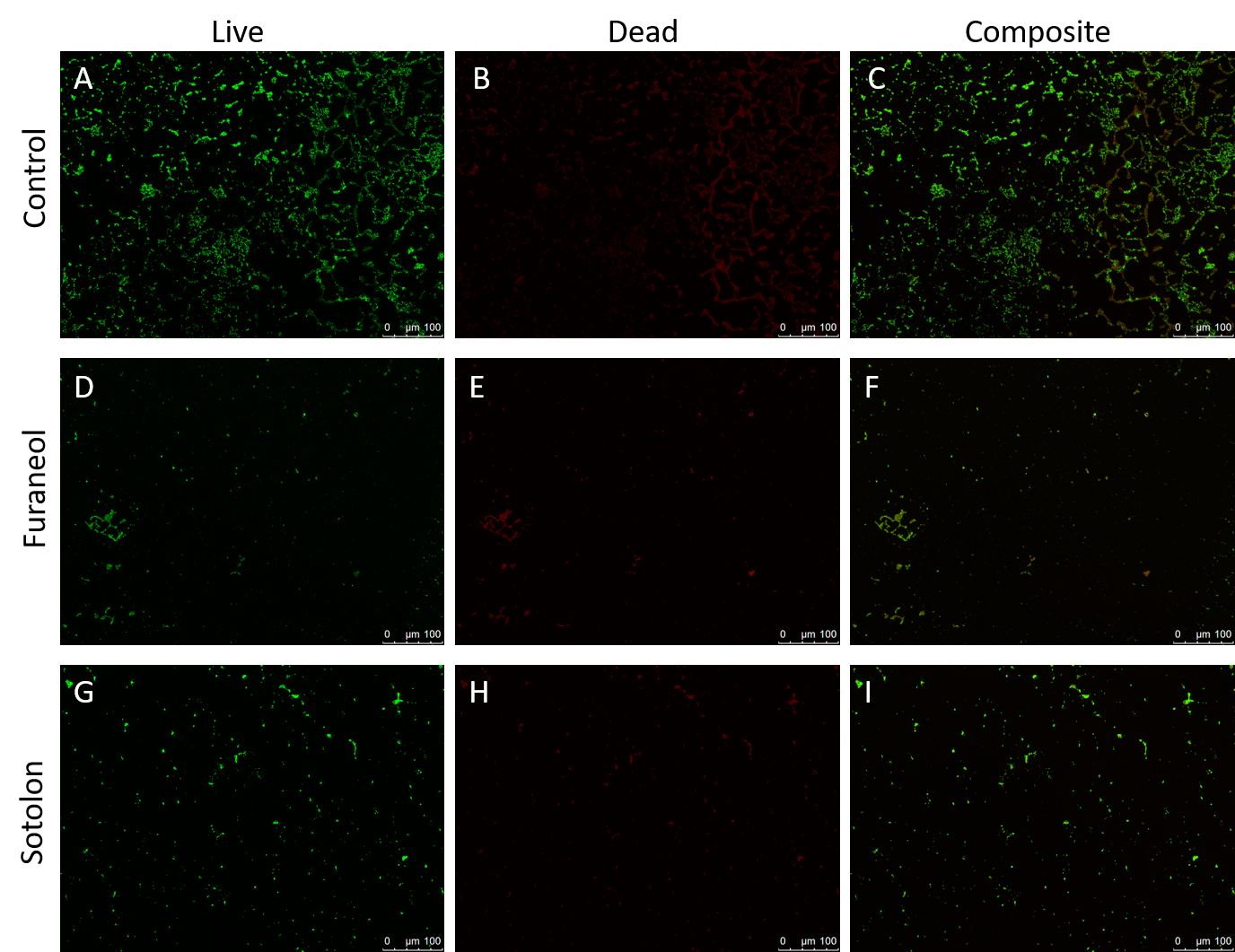


Supplementary Figure S6 – Baclight Live/Dead staining of established biofilms treated with furaneol or sotolon. When biofilms are left untreated (A-C), they appear well populated with good coverage of the growth substrate with a mix of both live and dead cells. When treated with 4 mg mL^-1^ furaneol for 24 h, biofilms become more sparsely populated (D-F). When treated with 4.33 mg mL^-1^ sotolon, biofilms appear similarly sparse. Images are representative of three independent replicates.


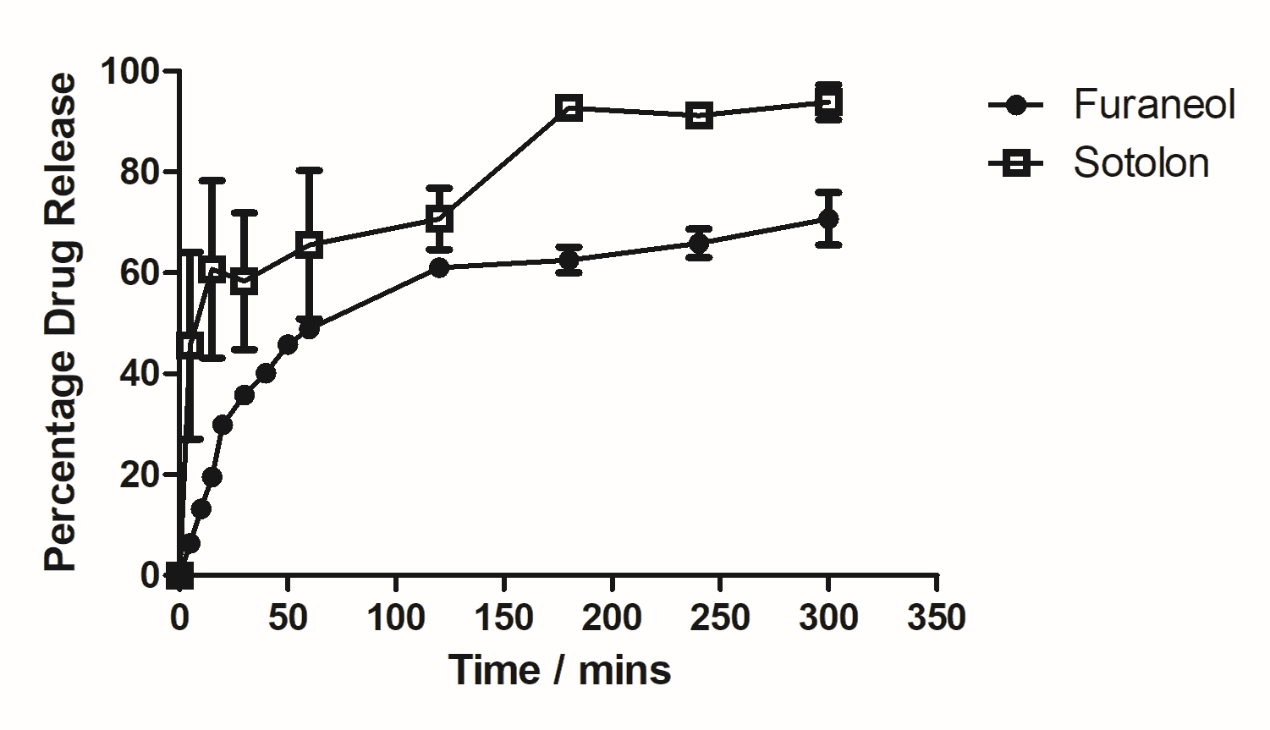


Supplementary Figure S7 – Release curves of furaneol and sotolon from a PVA aerogel. Furaneol loaded aerogels released 70.75% of their total loaded drug in a controlled manner over 300 min. Sotolon loaded aerogels released 93.85% of their total loaded drug over 300 min, with initial burst followed by more controlled release. Data shown represents the mean of three independent replicates (± S.D.).
